# Network analysis and site selection for cross-disciplinary collaboration: The Plant and Environmental Science Building at Michigan State University

**DOI:** 10.1371/journal.pone.0336032

**Published:** 2025-11-13

**Authors:** Felichism Kabo

**Affiliations:** Research Practice, CannonDesign, Ann Arbor, Michigan, United States of America; Queensland University of Technology, AUSTRALIA

## Abstract

Site selection is a complex, multicriteria process and a key if poorly understood contributor to the success of new scientific facilities. Typical site selection factors include the budget, sustainability, accessibility, utility and infrastructure costs, and environmental factors, e.g., air quality. Ideally, site selection should also reflect the organizational imperatives that drive the construction of new facilities in higher education, such as stimulating cross-disciplinary scientific collaboration. Traditional site selection is ill-equipped to account for the complex configurational effects of the built environment on cross-disciplinary scientific collaboration. Systems approaches, like network analysis, enable the simultaneous examination of a system (e.g., built environment) and its parts. This paper reports on a novel application of network analysis to examine the potential for site selection for cross-disciplinary collaboration for the Plant and Environmental Science Building (PESB) at Michigan State University (MSU). The network analysis of the two potential PESB sites, A and B, helped to identify which of the two sites is better configured to foster connections within PESB’s future research community. The results indicate site A’s paths are less deep on average and have more connections between other pairs of paths. Site A’s network is also more cohesive and less fragmented. Site A is thus better configured to support potential encounters and has a higher spatial legibility which correlates with better cognitive maps for individuals. These findings are all positively associated with collaboration. The study illustrates that network analysis can enable site selection that accounts for key organizational imperatives like cross-disciplinary collaboration.

## Introduction

The research ecosystems in public universities in the United States are facing difficult and uncertain budgetary environments with respect to funding for new scientific facilities, and leaders are increasingly expected to do much more with significantly less [1]. This is part of a broader phenomenon in OECD countries where the rate of growth of public research spending has declined sharply over the last three decades [2]. In this environment, universities face increased pressure to ensure that new scientific buildings are not only cost-effective but also meet the dynamic demands of their research and education missions. The placement of new scientific buildings within a university campus has non-negligible impacts on their occupants with respect to outcomes such as cross-disciplinary scientific collaboration [3]. Considering this, it is therefore noteworthy that the site selection process for new scientific buildings seldom incorporates cross-disciplinary scientific collaboration as a key decision criterion. This paper presents the study findings for a novel application of network analysis to examine the site selection process for a new scientific building at a major research university in the United States. While this specific case is a research-focused, interdisciplinary science facility the study has applicability to the site selection decision-making process for research and technology facilities in general. The work in this paper bridges a research gap on how the site selection process can incorporate organizational imperatives like cross-disciplinary collaboration.

There is compelling evidence that when it comes to cross-disciplinary collaboration, the spatial environment exerts significant influences. The research on spatial impacts on collaboration is robust as it spans disciplines, geography, and even straddles the COVID-19 pandemic [3–10]. Most studies, however, operationalize spatial effects using a distance decay function, based on the hypothetical premise that greater distances have negative impacts on complex social interactions and relationships, including collaborative ties (see, for example, [11, 12]). Spatial networks allow us to understand how position in space impacts collaboration outcomes. Further, spatial networks can yield critical insights into how the topological structure of a site or location shapes the social interactions that pertain to collaboration [4,13]. Moreover, site-specific network-level metrics enable us to compare the differences across sites or locations that likely impinge on interactions and collaborations among occupants and users.

Economic and real estate development professionals are keenly aware that site selection is critical to the future success and sustainability of any project or venture, including infrastructure projects such as buildings [14]. Where in economic and real estate development it is typical to optimize site selection with respect to a focal project or building, for universities the typical approach is to fit new buildings into an existing campus master plan. This approach in higher education privileges the larger scale of the entire university rather than individual buildings [15–17]. But in the context of declines in funding for scientific facilities, it means that there are higher costs to the type of failure that results from improper site placement of a building or facility. Simply, there are fewer financial resources to allow a “do over” and especially in the short- and medium-term. Rather, given the increasingly limited opportunities to construct new scientific buildings, institutions of higher education must hit the proverbial “home run” when it comes to optimizing for desired organizational outcomes like interdisciplinary collaboration. Over the last five decades, there have been relatively fewer opportunities for colleges and universities to construct new scientific buildings, especially when compared to the first three decades following the Second World War. This situation is related to the broader funding situation for higher education in the United States and other western nations.

The limited availability of funding for infrastructural spending in higher education [1] has been fueled by larger forces such as economic downturns, demographic shifts (hence declining enrollments), and shifting priorities among governments [18–20]. Unfortunately, the increasing scarcity in resources for constructing new scientific buildings coincides with the pressing need to replace aging infrastructure, some of which was constructed in the 1950s. This is a time when federal funding for scientific infrastructure as a percentage of all federal R&D funding was roughly three to six times higher than it is today [21].

### Funding for the construction of scientific facilities

In the United States, there has been a downward trend in government funding for higher education and public research. Economic downturns and shifts in state and local government priorities have led to a gradual, long-term reduction in spending on higher education (including capital projects) in favor of priorities like primary and secondary education, and healthcare [19]. for example, the revenues that public higher education institutions received in 2021 from state and local governments were down 80% from their levels in the 1980s [22]. These structural changes in higher education funding have significant ramifications for infrastructural investments related to research. For example, capital projects are complex, require coordination and consensus building with many stakeholders, and often call for years of planning, fundraising, and construction. The difficult funding regime means that many public universities have a shortage of the facilities needed to fulfill the research mission that society expects of them. The impacts of this shortage are felt not just in the quality of the faculty and student academic experience, but also in the research productivity of public universities [23].

**Fig 1:**
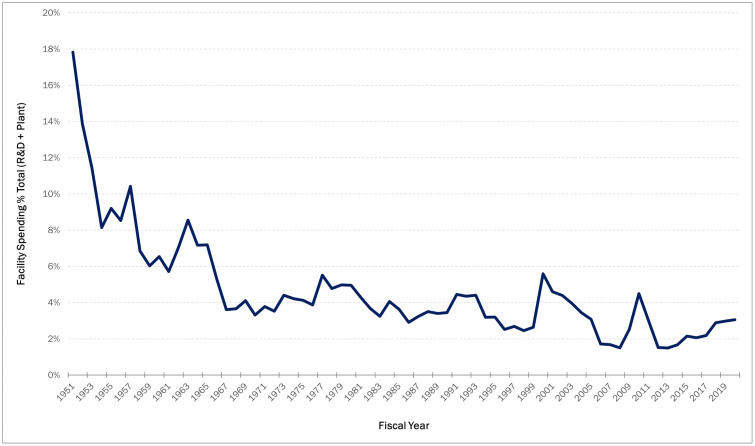
Federal funding for R&D plants as a percentage of total R&D spending, 1951 to 2020 (source data from: 21).

While more positive than at the state and local levels, the effect of the sluggish growth in federal funding for research and development (R&D) has inevitably trickled down to academic institutions. In FY 2020, R&D spending by academic institutions totaled $86.4 billion [24]. This was a $2.7 billion (3.3%) increase from FY 2019, which was the slowest growth since the 4 years of declining federal funding from FY 2012 to FY 2015 [24]. The long-term trend shown in Fig 1 indicates a steady decline in federal funding for R&D facilities as a percentage of all R&D funding starting in the 1950s followed by a more recent flattening in funding. This is even accounting for the bumps in federal R&D spending around the bursting of the dot-com bubble in the early 2000s, and the Great Recession of 2007–2009. For example, $457 million was spent on R&D plants in 1957, accounting for just over 10% of the total federal R&D spending of $4.4 billion. Fast forward 60 years to 2017 and $2.7 billion was spent on R&D plants, but this only accounts for roughly 2% of the total federal R&D spending of 121.6 billion.

Unexpectedly, the general trend in the decline or stagnation in federal funding for R&D facilities has not spared higher education institutions. Data from the *Survey of Federal Funds for Research and Development* [21] for the years 2018–2020 (Table 1) shows a pre-Covid 17% drop in federal R&D plant spending for universities and colleges, and a 6% reduction in funding for university-administered federally funded research and development centers (FFRDCs).

**Table 1:** Federal obligations and outlays for R&D plants, FYs 2018−20 (dollars in millions).

Performer	Preliminary			
	2018	2019	2020	Δ (2019–20) (%)
Total	3,852.7	4,376.4	4,759.0	8.7
Intramural	1,252.1	1,092.7	1,652.0	51.2
Industry	310.9	354.0	207.5	−41.4
Industry-administered FFRDCs	214.2	428.3	350.3	−18.2
Universities and colleges	410.4	514.0	427.1	−16.9
University-administered FFRDCs	761.5	850.0	799.3	−6.0
Other nonprofit institutions	180.2	169.8	241.9	42.4
Nonprofit-administered FFRDCs	709.4	956.2	1,076.3	12.6
State and local governments	0.0	1.2	1.0	−15.0
Foreign	14.1	10.3	3.5	−65.8

The fewer resources available for the construction of new facilities means there is added pressure on leaders and administrators in higher education to “get it right” in terms of fostering the multi- and inter-disciplinary science that attracts funding, faculty talent, and the best students. While universities place high hopes on the diminishing pool of new science buildings to foster cross-disciplinary scientific collaboration, site selection is a potentially serious blind spot in the process of building these new facilities.

Site selection is a complex, multicriteria process involving many factors and stakeholders [25]. Typically, for new science buildings at universities, this process is driven by project requirements and criteria including the budget, land size, topography, sustainability, accessibility, utility and infrastructure costs, and environmental factors such as air quality and solar radiation. Often unaccounted for are the key outcomes of primary interest to the university, such as fostering cross-disciplinary scientific collaboration across academic units. These scientific outcomes are contingent on both planned and unplanned encounters and interactions among scientists which are shaped to various degrees by the topology or configuration of the built environment. The complex and dynamic topological effects of the built environment shape collaboration at multiple levels and scales, from that of the individual workspace to that of the entire campus.

Traditional approaches to site selection are inadequate for incorporating the imperatives for collaboration into the site selection process as they are reductive in that they treat complex, intertwined factors in isolation [26]. In contrast, systems approaches, such as network analysis, enable the simultaneous examination of a system and its parts. In this paper we present the findings from a research study that applied network analysis to examine site selection for collaboration at a new science facility at a major research university in the United States.

### Literature review

#### Current approaches to site selection.

Site selection is an example of a spatial multicriteria decision making process that entails the evaluation of multiple criteria according to often contradictory objectives [27,28]. General multicriteria analysis is a decision-making and mathematical tool that gives the decision-maker the ability to compare different alternatives or scenarios given these often conflicting criteria so as to make an optimal or judicious choice [29]. Spatial multicriteria decision making is the application of multicriteria analysis in spatial contexts where key elements of the decision problem, for example, the alternatives or scenarios, criteria, etc. have explicitly spatial dimensions [28]. A spatial decision alternative or scenario is comprised of at least two elements [28]: an *action* (what needs to be done), and a *location* (where it needs to be done). Spatial evaluation criteria are associated with geographical entities, and the topological relationships between these entities [28]. These criteria are usually represented in the form of maps, and hence the use of geographic information systems (GIS) for spatial multicriteria analysis (SMCA) in a range of applications such as urban planning, retail, renewable energy, transportation, and resource management [28,30,31].

The site selection process is an integral part of the success of any building project. Key considerations during this process include financial and environmental factors. For example, selecting the wrong site for commercial buildings such as shopping malls can have negative upstream and downstream financial effects such as on the profitability of the investment [32,33]. For all projects, the site informs design decisions related to the building’s orientation, massing and configuration, landscaping, etc. [34]. Moreover, the site shapes the environmental comfort of the building occupants as it is directly associated with issues like access to clean air, water, and solar radiation [34,35]. Related to that, the immediate site and its environs have significant influences on factors that shape the health and safety of a facility’s occupants and users, e.g., crime, cleanliness, and tidiness [32,34].

The criticality of site selection is reflected in the increase in more advanced approaches to the process beyond the intuition or simple heuristics that are prevalent today. These include, for example, multiple-criteria decision-making (MCDM) methods such as the analytic hierarchy process (AHP) and the analytic network process (ANP) that provide structured techniques for organizing and analyzing complex decisions [36–39]. AHP is an Eigen value approach to pair-wise comparisons for quantitative and qualitative variables that helps decision makers arrive at the most logical choice from a set of alternatives [40]. An example of the application of AHP in site selection is in the assessment of market factors, environmental conditions, regulatory factors, and transportation to determine where to best place a hospital from among 12 districts in the Mugla province of Turkey [41]. The ANP is considered a generalization of the AHP to feedback networks [42,43]. The AHP structures a decision problem as a hierarchy with three types of elements: a goal, decision criteria, and a set of alternatives. Each of these elements can be viewed as independent of the others. In contrast, the ANP allows dependence among the elements, and by treating them as a network also allows for feedback between the elements [44,45]. Thus, the ANP has been proposed as a solution to the problem of selecting the best site for a shopping mall [32], though its methodology maintains generalizability across facility types. This process entails an examination of interdependent criteria such as transportation for users, total investment costs, environmental considerations, and development potential.

As previously noted, the use of geographic information systems (GIS) to drive or support the site selection process has grown in prominence, especially over the past two decades. GIS has been defined as the “organized activity by which people measure and represent geographic phenomena then transform these representations into other forms while interacting with social structures” [46]. GIS is a system for understanding the fundamentals of issues unique to spatial data such as geolocation, proximity, and distance decay [47–49]. GIS-based approaches enable the combination of spatial data such as street networks with non-spatial data such as demographic information related to population and economic activities [50]. Major applications of GIS include urban planning [51], transportation analysis [52], public health and epidemiology [53,54], effects on the built environment on health [55], and crime analysis [56]. Of higher relevance to this paper is the use of GIS in location analysis [31], including for specific types such as shopping malls and other retail facilities [57,58], industrial sites [27], and wind turbine farms [30]. There have also been efforts that combine GIS with, for example, Building Information Models (BIM) for both site selection and fire management processes [59].

More recently, there has been a rise in the use of data-driven approaches to site selection. Traditional approaches have several limitations, such as limits on the complexity of their models, and a reliance on expert or subjective judgments. Data-driven approaches are better suited to accounting for the complexity of site selection as a decision-making process. For example, supervised machine learning was used to address the retail store placement problem using geographic and human movement data from location-based social networks [60]. Another data-driven approach to the retail store placement problem used search query data mined from Baidu Maps, China’s largest online map search engine, to predict and rank the number of customers [61]. These data-driven approaches enable more complex models of the site selection process compared to traditional approaches. However, in common with traditional site selection, data-driven approaches still employ a reductive approach that makes them ill-suited for site selection based on complex, organizational outcomes. For example, cross-disciplinary scientific collaboration among scientists at a university is often contingent on the relationships among the buildings (and their elements or components) on campus.

We report the results of a novel application of network analysis to examine the structure of the complex systems comprised of the interconnected floors and buildings at two potential sites for the PESB on MSU’s campus. We then perform analysis to identify which of the two sites has the better structure or configuration for supporting cross-disciplinary collaboration, one of the key imperatives for the PESB.

### Site selection & network analysis

Reductive approaches to site selection are ill-suited for the task of site selection for cross-disciplinary collaboration in the context of new scientific facilities. Understanding the configuration of the paths or walking routes interconnecting buildings on campus and how they shape the potential encounters and interactions associated with cross-disciplinary collaboration among scientists calls for a systems approach, such as network analysis. In common with other systems approaches like agent-based modeling, network analysis is both a theoretic perspective and set of methods that considers the interrelationships between the components of a system as opposed to focusing on isolated parts of the system, as well as the system itself. Moreover, network analysis is agnostic about levels of analysis, meaning that we can also examine systems of systems. For example, a graph convolutional network (GCN) built using geospatial and transit data in Singapore revealed the spatial interaction patterns of different locations on a map and, subsequently, their impact on store placement [26].

Prior research has identified factors of the built environment that are more supportive of collaboration in knowledge work, including multi- and inter-disciplinary science. Several of these factors relate to the proximity effects resulting from the configuration or layout of buildings and places and are thus amenable to studies employing the network approach. These include, for example, orienting key spaces such as labs and offices to optimize overlaps in the paths taken by investigators as this is correlated with the potential or chance encounters that are essential to collaboration and innovation [5,6]. Another factor is the minimization of the topological (turns in the shortest paths) and physical distances between the members of a potential collaboration dyad [3,4]. Finally, the built environment is more amenable to supporting collaboration when paths or walking routes between potential collaboration dyads have shorter topological distances (fewer turns) as this results in space that is less fragmented [13].

To the author’s knowledge, there are currently few if any examples in research or practice where network analysis has been employed as an analytic or planning tool for new scientific facilities in higher education. This is a significant gap considering the importance and benefits of proper site selection for new scientific buildings in the context of shrinking resources for facility construction across the higher education landscape in the United States. For the science of site selection, a key research gap is the absence of methods that simultaneously account for spatial configuration and the potential social network dynamics related to site selection, especially the drivers of organizational outcomes like collaboration, innovation, and interdisciplinary science. The approach adopted in this study, spatiosocial network analysis, integrates spatial and social network metrics to examine how spatial configurations can support social processes like cross-disciplinary scientific collaboration, rather than just optimizing site selection for isolated criteria.

### A new approach: Spatiosocial network analysis and visualization

This study uniquely combines spatial and social network analyses and visualization to examine the configurational properties of two potential sites for a new scientific building. The spatial networks formed by the connections between discrete units in a building, campus, or city are generally planar because both their vertices (the discrete units) and edges (the connections between the units) are fully spatially embedded [62]. This means the networks can be represented on a flat surface without any of the edges between the discrete units overlapping or crossing each other [63]. In contrast, non-planar spatial networks can include spatially embedded vertices with overlapping edges that represent non-spatial relationships such as social relationships, telecommunications, etc. [62]. Finally, some spatial networks have both planar and non-planar characteristics, like travel routes [62]. A key advantage of the approach outlined in this paper is that tools used for social network analysis can be used to powerfully visualize complex planar and non-planar spatial networks to better identify patterns, clusters, and anomalies within the network. Even for the planar networks formed by discrete units in spatial systems like buildings and campuses, these visualizations can provide deeper insights into the structure and dynamics of the system’s network. This is essential when making comparisons between potential sites for the location of a new scientific facility.

The organization of social and economic activities in human societies is often contingent on the act of configuring continuous space into discrete units that can then be labeled, assigned to specific individuals and groups, and so on [64]. This reality is the point of departure for the approach to spatial network analysis known as “space syntax” which was first developed in the 1970s and has since been used to examine relationships between spatial layouts, and social, economic, and environmental outcomes at multiple levels from building interiors to campuses, cities, and regions [65–67]. As a theoretic perspective and computational approach, “space syntax” was intended to help architects employ science-based, human-focused spatial network analysis that could better simulate the effects of design solutions on occupants and users [68]. For the site selection process, crucial insights can be gained from the examination and comparison of “space syntax” whole network measures across different sites. Key whole network measures help us understand not only how spaces or discrete units are connected, but also how the movement of people flows through them. While these measures have thus far not been applied to the critical task of site selection, the analysis of the measures has been used by architects, planners, and designers to identify key areas for improvement, optimize spatial layouts for better movement and interaction, and understand the social dynamics of different built environments. This approach helps create more efficient, safe, and vibrant spaces that are better tailored to human needs.

Social network analysis (SNA) is a theoretical perspective and set of methodologies that are used for the empirical study of the patterns of relations among actors in social groups and collectivities, including the structure of relationships among actors at different levels of analysis [69]. A network is defined as consisting of the actors which are variously defined as “vertices” or “nodes, and the relationships among the actors which are referred to as “edges” or ”ties.” The network perspective has transformed the social sciences over the last half century, especially in tandem with advances in network methods and the computing power needed to model and study large, complex social networks [70]. Network theory provides powerful explanations for a range of important social phenomena ranging from individual-level outcomes like creativity to group-level outcomes like innovation and profitability [71]. SNA provides valuable insights into how information, resources, and influence flow within a network. The potential applications of these insights include helping to identify key players, potential bottlenecks, and opportunities for intervention or improvement. The network perspective has significantly advanced our understanding of the mechanisms of scientific collaboration and innovation in the social structures of academic campuses. For example, a study showed that spatial proximity and the sense of community (SOC) construct have potentially complementary effects in the network positions of scientists where only some of them were co-located [72]. Another study found that institutional affiliation and spatial proximity were significant drivers of collaborative communities in longitudinal scientific networks [73]. Similarly, a study of collaboration patterns at the Massachusetts Institute of Technology (MIT) found that the network topology and community structure were biased by spatial configuration and institutional affiliation [3].

The combined application of spatial and social network analyses and visualization is a potent approach to understanding differences across potential sites both empirically and visually.

## Methods

### Study site and timeline

**Fig 2:**
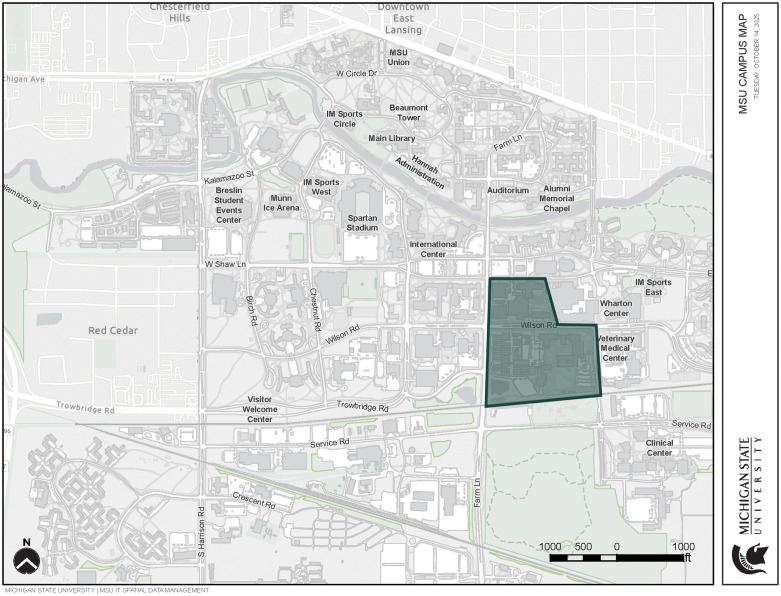
MSU campus map with the area comprising of the existing plant sciences neighborhood and the Biomedical and Physical Sciences Building shown in the shaded green polygon (source: 77).

This study focuses on the application of network analysis to examine the collaboration promotion potential of two sites that were considered for the new Plant and Environmental Science Building (PESB) at Michigan State University (MSU). The two potential PESB sites are in the area adjacent to the existing plant sciences neighborhood and the Biomedical and Physical Sciences Building on MSU’s campus in East Lansing, Michigan (Fig 2).

The $195 million PESB was conceived with a broad, ambitious scope. It ranges from addressing capacity issues, attracting and retaining leading scientists and top students, increasing federal research funding, and expanding the critical agricultural research that supports the state of Michigan and the United States [74]. By collocating top scientists at the cross-disciplinary intersection of plants and the environment, and placing them in a neighborhood of biomedical and physical scientists, PESB is intended to promote synergistic collaborations and new discoveries [74]. The decisions around PESB’s potential site locations reflect Vision 2050, MSU’s integrated facilities and land use plan, and infrastructural requirements [74,75]. That is, in line with Vision 2050, PESB’s location had to be one of the two potential locations examined in this study. This study, therefore, played no role in MSU’s final site decision. Rather, the study examines how the PESB site selection process could have been done to better account for the organizational imperatives of stimulating cross-disciplinary collaborations and discoveries. PESB’s construction will start in October 2024 [76].

The study described in this paper is part of a broader effort to understand the longitudinal, spatiosocial impacts of PESB on outcomes related to collaboration, innovation, and translation. This study’s narrower focus on site selection for PESB can advance our understanding of how this process can be informed by a key organizational imperative for MSU, stimulating cross-disciplinary scientific collaboration. As noted, one of the biggest drivers for site decisions around PESB was the strategy outlined by MSU’s integrated facilities and land use plan. This paper focuses on the simpler issue of using network analysis to examine the potential of site selection for outcomes like cross-disciplinary scientific collaboration [77].

**Fig 3:**
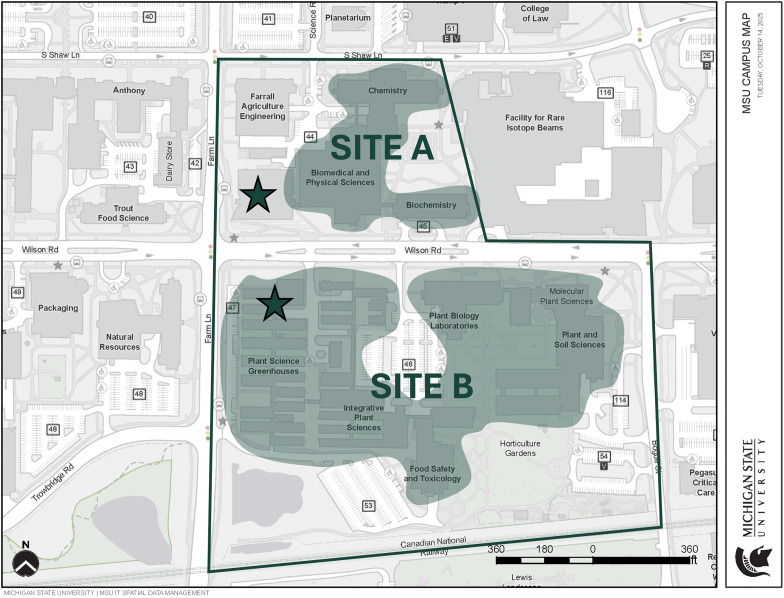
Map of the plant science neighborhood showing the two potential sites straddling Wilson Road. **(basemap source: 77).** The shaded areas overlay the buildings that are used to generate the spatial network for each site. The stars represent the proposed locations of PESB at that specific site.

The data collection and analysis period was from March 2023 through June 2024. Work on earlier versions of the manuscript commenced in December 2023. Following a conference presentation in June 2024 and additional analysis from July to October 2024, work on the final manuscript started in October 2024 and was completed by March 2025. The two potential sites (A and B) for MSU’s PESB are part of a much larger plant sciences neighborhood that is bifurcated by Wilson Road (Fig 3). Vision 2050 identifies this neighborhood as part of the campus precinct named “Central Campus” which has been targeted for investments that expand MSU’s academic and research missions [77]. Our study focuses on the buildings in this neighborhood that house current and potential members of the research community that will be relocating to PESB. Note that data collection in March 2023 consisted of obtaining the electronic floor plans for the buildings that were used to generate the spatial networks for the two sites from MSU’s Infrastructure Planning and Facilities (IPF) organization. The population of interest are the investigators that are slated to move into PESB upon its completion. Specifically, we examine two potential sites for PESB with respect to which of them is better placed to facilitate the encounters and connections among the investigators that correlate to cross-disciplinary scientific collaboration. The investigators are all part of an existing research community primarily consisting of the plant sciences, but also spanning multiple other disciplines including biochemistry, chemistry, biomedical sciences, and physical sciences. Members of this research community are also currently collocated across multiple buildings in a larger neighborhood that encompasses the two potential sites for PESB.

Site A is north of Wilson Road and has three buildings that are interconnected at the basement level, and by a ramp spanning the fourth and fifth floors. These focal buildings are fringed by (but not connected to) three buildings that house different research communities from our study population, with plant sciences at its core. Site B is south of Wilson Road and comprises five buildings, most of which are interconnected at the basement, first, and second floors. This site is also where the majority of future PESB occupants are currently located. Site B also has support facilities such as greenhouses and a headhouse.

### Spatial network generation

For each of the two potential sites, we constructed a spatial network based on paths or lines of navigation and movement across all the buildings, also known as an axial map [65,66]. This represents the longest straight lines of sight and movement within a spatial system. Generating the axial map entailed drawing, in AutoCAD, the set of fewest and longest paths that made all possible connections between individual spaces and rooms, and between the buildings at the floor levels where they were interconnected [78]. For each building, staircases are treated as inter-floor connectors and are represented by a dummy space during the generation of the spatial network. This raw shapemap was then exported from AutoCAD to the depthmapX software application where it was converted into an axial spatial network [79]. The spatial network for each site therefore represents all possible connections between spaces or rooms, floors, and the buildings housing current and potential members of the PESB research community. The axial spatial networks have several variables associated with them that measure the local and global properties of a specific path’s location in the spatial network. In this paper we will focus on a couple of these whole network variables or measures to illustrate key differences in the complexity of the differences in spatial network structure across the two sites.

### Visualizations from social network analysis

It is important to visualize the spatial networks for the two sites such that it is easier for individuals without spatial training to visually identify the configurational differences between the two sites. People who lack spatial training, including many social scientists, may find it difficult to read and interpret diagrams like floor plans and blueprints, and hence the axial networks described above. Lower levels of spatial training correlate to lower levels of spatial intelligence, the ability to visualize and manipulate objects in a three-dimensional space, which is crucial for understanding these types of diagrams. Unsurprisingly, research has found that there are significant differences in spatial intelligence even among advanced and beginner architecture students [80]. Further, it is important to account for the fact the spatial network metrics from “space syntax” are largely unfamiliar to most scientists who study scientific collaboration and innovation. Therefore, to better communicate the topological differences across the two sites, it is helpful to generate the types of measures associated with social network analysis as this are currently better understood by a wider cross-section of scientists from the social and physical disciplines. Undertaking this approach enables both visual and empirical comparisons of the two sites in ways that are easier to understand for a broader audience.

Therefore, we exported the axial maps from the spatial networks platform to social network analysis software applications in order to generate the types of network graphs familiar to those from the social and physical sciences [79,81,82]. We subsequently used these graphs to generate a set of whole network variables that are described in the section to follow. This process enhanced the empirical and visual comparisons of sites A and B with respect to their capacity for supporting potential encounters and interactions before the addition of PESB.

### Variables

From the axial spatial networks, we generated two primary whole network variables. Note that these variables are global in the sense that they capture a focal path’s relationship to all other paths in the spatial network, rather than to only a part of the network. “Mean Integration” is the average value of the measure of integration for all the paths, which is defined as the average depth of the path from all other paths across the site. This measure shows how easily accessible a space is from all other spaces in the network. High integration values suggest central, well-connected areas that are likely to attract more activity. “Mean Choice (Normalized)” is the average value of the choice measure for all paths across the site and captures the frequency with which the path lies on the shortest connections between other pairs of paths at the site. This measure is normalized to account for the size of the spatial network (number of nodes), thus making it possible to compare our two sites. The choice measure reflects the likelihood of a space being used as a route between other spaces. High choice values indicate spaces that are crucial for through-movement and can highlight important pathways or corridors, and the areas where serendipitous or chance encounters are most likely to happen.

Using the social network graphs, we computed nine variables for comparisons of the spatial networks for the two sites as follows. “Number of nodes” captures the number of paths in all the buildings at the site, while “Number of ties” represents the connections between these paths. The geodesic distance is the shortest path between two nodes in the network. The “Diameter” represents the length of the largest geodesic distance in the network. The “Average Distance” is the mean geodesic distance for all pairs of nodes that are reachable in the network. Degree centrality is a network measure of the number of direct connections that a node has. “Average Degree” is the mean degree for all the nodes in the network. “Components” captures the number of strong components which are subsets of the graph where all the nodes are directly connected to each other. “Fragmentation” equals one minus the proportion of nodes that can reach other nodes by any path. “Transitivity” measures the likelihood that two nodes with a connection in common are also connected to each other. The “Proportion within three” measure indicates the proportion of pairs of nodes in the network that are within three steps or ties of each other.

To better understand how the two sites differed with respect to the structure of their networks, we also computed the variable “Δ” which measures the percentage difference in the values of the network variables above as shown in Equation 1 below:


$$\Delta =\;\left(\left({Value}^{Site\;A}-\;{Value}^{Site\;B}\right)÷{Value}^{Site\;A}\right)\;\times \text{1}\text{0}\text{0}$$ (1)

Understanding how the two sites vary across the network variables described above can help paint a clearer picture of what aspects of spatial network structure should be considered during the site selection process. Importantly, this entails examining the implications of the spatial network differences across the two sites with respect to potential encounters and interactions among the occupants of the buildings on each site and, subsequently, the resultant impacts on cross-disciplinary collaboration.

### Research questions

In the sections to follow, we will focus our discussion of the empirical differences between sites A and B on these whole network metrics. This discussion assumes that network analysis can help us identify which of the two sites is better configured for the proximity effects that facilitate potential encounters and interactions among investigators. It is presumed that this site would be the better location for the future PESB building given the key organizational goal of stimulating cross-disciplinary scientific collaboration.

The previous section on site selection for collaboration using network analysis identifies several factors that render a site more likely to support cross-disciplinary scientific collaboration. These factors are operationalized by multiple variables in this study and will be examined via addressing three research questions which relate to the network variables diameter, average topological distance, and fragmentation. Network diameter, the longest shortest path between any two nodes in a network, reflects the maximum extent of connectivity and is a good indicator of movement efficiency and reachability across the site, key factors in cross-disciplinary collaboration. The average topological distance (or average path length) is the mean number of steps required to traverse the site. A lower value would therefore suggest a more integrated or navigable site, which could potentially facilitate more chance or unplanned interactions, and hence cross-disciplinary collaboration. Lastly, fragmentation captures the extent to which the site is broken into disconnected or weakly connected components. Lower fragmentation would indicate a more cohesive site, which would ease movement, and enhance accessibility and interaction, thus making the site more likely to promote cross-disciplinary collaboration. The three research questions are:

- **RQ1** – is there a difference in the network diameter between the two sites?
- **RQ2** – is there a difference in the average topological distance between paths or walking routes at the two sites?
- **RQ3** – is there a difference in the fragmentation of the spatial network of paths or walking routes at the two sites?

In the next section the study findings are used to discuss the research questions above and to compare the two sites to identify which of them is more amenable to supporting scientific collaboration.

## Results

### Spatial network graphs

**Fig 4:**
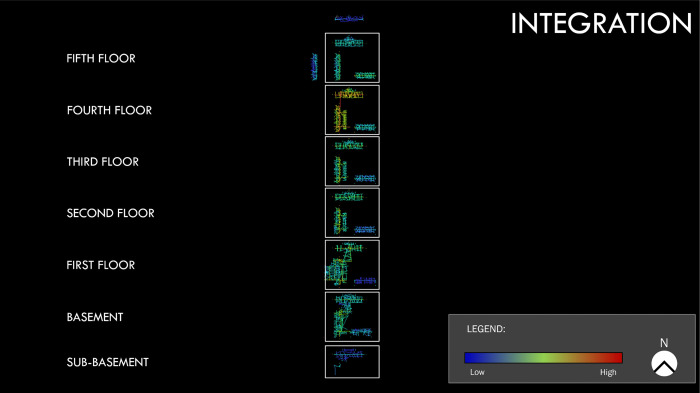
Site A axial spatial network for the measure of integration, which captures how central or easy it is to access the path of movement or navigation across the entire site. Map colors indicate path centrality as shown on the legend with paths coded blue having low integration values and those coded red having high integration.

Figs 4 (site A) and 5 (site B) show the axial spatial networks for the two sites. The networks have been rendered to better illustrate which paths of movement or navigation are most central in the spatial network for all the buildings in the site’s network. That is, the paths of movement or navigation in each axial spatial network are presented such that we can visually identify which ones are the most central across the entire site or spatial system.

**Fig 5:**
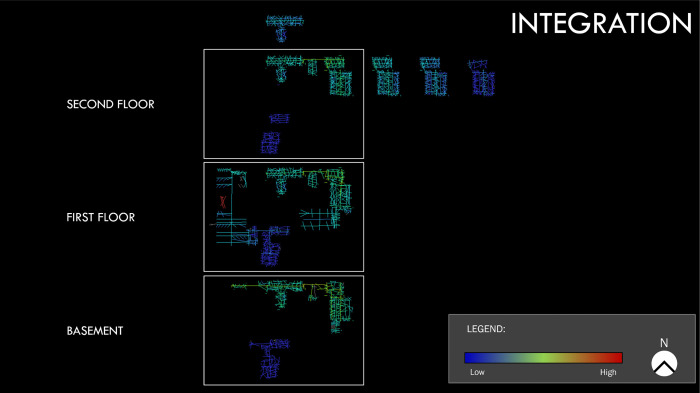
Site B axial spatial network for the measure of integration. Map colors on the legend indicate path integration values where the cooler the color is, the lower the integration value, and the warmer the color is, the higher the integration value.

### Social network visualizations

**Fig 6:**
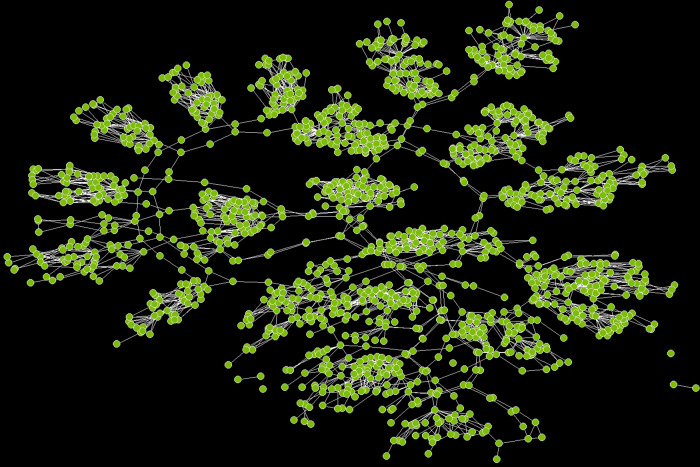
Network graph for site A. The graph for site A reveals that, as is the case with many real-world planar networks where local connectivity is crucial, there is high clustering in the network. However, there is also visual evidence for path redundancy and numerous cycles which enhance this network’s robustness and failure tolerance as it suggests that there are multiple ways of traversing the network without retracing one’s steps. These path-based characteristics of the network facilitate more efficient communication and information flows across the site.

**Fig 7:**
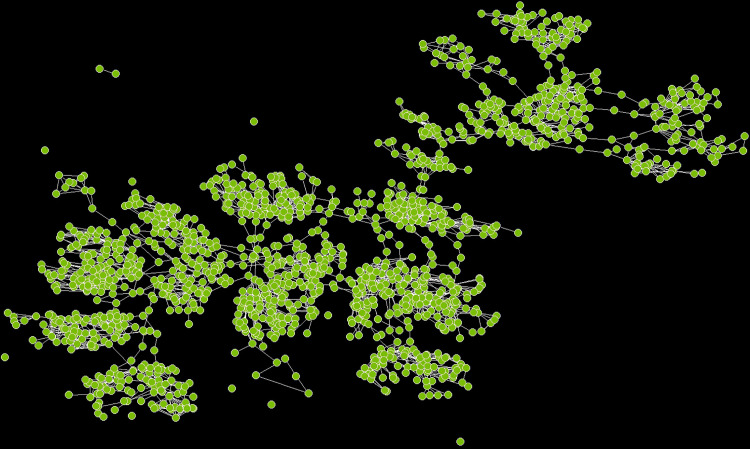
Network graph for site B. The graph for site B exhibits high clustering as one would expect for this type of spatial network. Compared to site A, however, this site shows evidence for limited path redundancy and fewer cycles. There are fewer alternative paths between nodes (spaces) making the network less robust to failure, and more limited ways of traversing the network and returning to the starting point without retracing one’s steps. These characteristics can negatively impact the site’s efficiency and overall connectivity.

Figs 6 and 7 represent the types of graphs that are more familiar to network scientists. These graphs enable us to make visual examinations of how the two sites vary with respect to concepts associated with the network’s connectivity such as clusters (groups of nodes that are densely connected to each other), paths (sequences of nodes with no repetition) and cycles (closed paths starting and ending at the same node). Site A has cutpoints (the narrow-waisted region) that make it more vulnerable than site B which, in contrast, has more cycles or cyclic loops. Thus, site A is like real optimal networks in nature, such as leaves or insect wings, which have a high density of cyclic loops that increase resilience to attack and damage [83].

### Whole network metrics

The combined metrics from the whole network analysis of the networks in Figs 4–7 are shown in Table 2 below. Recalling that the fourth column shows the percentage difference in the value of a network variable between the two sites. In the interest of brevity, we will limit our discussion of the results to those network variables where the variable Δ between the two sites is at least 5.0%.

**Table 2:** Comparison of sites A (*nodes = 1,562, ties = 5,408*) and B (*nodes = 1,617, ties = 5,834*) across select network variables.

Variable	Site A	Site B	Δ (A-B) (%)
Diameter	31	40	−29.0
Average Distance	13.392	16.415	−22.6
Average Degree	3.462	3.608	−4.2
Components	8	22	−175.0
Fragmentation	0.011	0.036	−227.3
Transitivity	0.151	0.237	−57.0
Proportion within three	0.029	0.024	17.2
Mean Integration	0.650	0.523	19.5
Mean Choice (Normalized)	0.010	0.008	25.5

The two sites have roughly about the same number of nodes (paths) though site B has nearly 8% more ties or connections between its nodes. Site A has both a smaller diameter and average distance, indicating lower costs for individuals at site A with respect to the effort of physically seeking out others at the site.

With respect to the research questions, site A has a network diameter of 31 which is 29% less than site B’s. The results provide support for RQ1, that there is a difference in network diameter between the two sites. Site A’s lower network diameter also indicates higher connectivity in its spaces and buildings compared to site B.

The results indicate that there is an average distance of 13.392 between the paths or walking routes at site A, which is 23% lower than at site B. Therefore, the results provide support for research question RQ2. Moreover, these results suggest that movement and navigation is more efficient at site A, and that its path network is more interconnected than site B’s.

From the results presented in Table 2 we can conclude that there is less fragmentation at site A. Specifically, site A has a fragmentation of 0.011 which is 227% lower than site B’s. This indicates that the paths within site A have many connections among them compared to those at site B, and that there is therefore a higher level of connectedness at site A. Thus, the results provide support for research question RQ3. The results can be interpreted to mean that at site A it is easier to reach any path or walking route from any other path than at site B.

The axial spatial network measures in Table 2 reveal additional ways in which paths differ across the two sites. First, they indicate that the typical path on site A has a higher integration value which means that it is less deep on average from all other paths in its spatial network compared to the typical path at site B. Second, the typical path on site A has a higher normalized choice value, indicating that it is likely to have more connections between other pairs of paths passing through it than the typical path at site B. This finding implies that the typical path at site A is more likely to have a higher potential flow of movement, and hence a higher likelihood of potential encounters between the people who occupy the buildings at the site.

The results shown in Table 2 indicate that the nodes in site A’s spatial network have more cohesion than those in site B’s network. In network terms, the site A network is less cliquey than site B’s, as shown by the fact that it has fewer components and lower transitivity. The results also show a lower fragmentation for site A, giving us more evidence that the paths (and spaces) in site A are more tightly connected within the site than is the case for site B. The smaller number of strong components in site A compared to site B shows that the former has low clustering as components can have weak ties to other parts of the network, even as all the nodes in the network have strong ties with each other. Coupling site A’s lower average distance with its higher proportion of nodes that are reachable within three steps suggests higher connectivity and efficiency compared to site B, as pairs of nodes or paths in site A’s spatial network are reachable in fewer steps. Overall, the results indicate that site A’s configuration can engender more potential encounters and interactions than site B, as it is better connected and more efficient with respect to movement and navigation across all spaces, floors, and buildings.

## Discussion

The study findings provide support for the research questions regarding the differences in network characteristics between site A and site B. First, the network diameter of site A is lower than that of site B. This supports RQ1, indicating that site A has higher connectivity within its spaces and buildings compared to site B. A lower network diameter suggests that any two points within site A are closer together, facilitating easier and quicker navigation. Second, regarding RQ2, the average distance between paths or walking routes at site A is lower than at site B. This finding implies that movement and navigation are more efficient at site A. The reduced average distance enhances accessibility and reduces travel time within the site. Third, the results also address RQ3 by demonstrating that site A has a lower fragmentation than site B’s. This lower fragmentation indicates a higher level of connectedness within site A, meaning that paths are more interlinked, making it easier to reach any path from another. This interconnectedness is crucial for efficient movement and accessibility.

Further analysis of axial spatial network measures reveals that site A has higher integration and normalized choice values. These metrics suggest that paths at site A are less deep from all other paths and have more connections between other pairs of paths passing through them. Thus, the results indicate that site A’s spatial network is more compact and better connected than site B’s. Consequently, site A is likely to experience a higher potential flow of movement and more frequent encounters among occupants. From the perspective of stimulating cross-disciplinary scientific collaboration, this has multiple advantages. Site A offers higher efficiencies with respect to potential travel times for movement and navigation. The smaller average distances that site A has can also facilitate the exchange and diffusion of information, resources, and even innovation. This has positive effects on accessibility across the site, and collaboration within the site.

Moreover, the cohesion of nodes in site A’s spatial network is greater than in site B’s, as evidenced by fewer components and lower transitivity. This indicates that site A is less fragmented and more tightly connected, enhancing overall connectivity and efficiency. The combination of lower average distance and higher reachability within three steps further underscores site A’s superior connectivity and efficiency. The lower fragmentation at site A coupled with the fact that it has fewer strong components means that there are more paths that intersect with each other across the site, making it more likely that site occupants will have serendipitous encounters with one another on a regular basis. This has significant implications for cross-disciplinary collaboration given the evidence for the importance of serendipity during different stages of the collaboration process [84–86]. The fact that the site A configuration has more paths that intersect also makes it more likely that there will be more overlaps in the functional paths taken by individuals on a regular basis. The greater the functional path overlap between a pair of scientists, the more likely it is they will collaborate [5]. Moreover, larger functional path overlaps between potential collaboration dyads are also associated with a better likelihood of success for scientists [5].

The other advantage site A has is with respect to the cognitive maps of the site occupants. Cognitive maps are mental representations of the spatial information needed to navigate and locate resources in our environment [87]. For our purposes, a cognitive map is also a person’s mental representation of the network that is acquired through exploration and navigation, over time. The topology or configuration of a spatial network has significant impacts on the accuracy of individuals’ cognitive maps [88]. And the more accurate one’s cognitive map, the easier their wayfinding and sense of spatial orientation [89], which affects their ability to locate others and resources in the spatial system. The legibility of a spatial system is the degree to which it aids people in creating effective cognitive maps [90–92]. The topology or configuration of a spatial system is a key objective factor that shapes its legibility due to its direct impacts on a person’s spatial cognition [90,91,93–95]. Higher integration values are associated with better spatial legibility [96] which, based on the study findings, suggests that site A should be more legible than site B, and that the former should also be associated with more accurate cognitive maps for its occupants. Prior research suggests that more accurate cognitive maps enable people to communicate and collaborate more effectively [97].

Generally, the findings imply that Site A’s topology or configuration is more likely to stimulate and support cross-disciplinary collaborations. For example, site occupants are more likely to have better or more accurate cognitive maps of the entire site including the locations of potential collaborators. The key drivers behind MSU’s decision to build PESB included stimulating scientific collaboration, retaining current members of the plant science community, and attracting new talent. It is therefore imperative that a site’s potential for supporting scientific collaboration should be one of the important factors that are considered during the site selection process. The network analysis described in this paper identified site attributes that are correlated with collaboration, and revealed that these factors were stronger at site A compared to site B.

## Conclusions

In an era of declining or stagnating federal funding for R&D facilities in higher education, it is critical that the design and construction of new facilities be in complete alignment with the primary objectives of higher education institutions. These include the need to stimulate cross-disciplinary scientific collaboration, which is directly correlated to the institution’s success in research and access to funding, and the recruitment and retention of talented faculty and students. Site selection is an integral part of every new building, including scientific facilities. However, organizational objectives such as the catalysis of cross-disciplinary collaboration are seldom, if ever, accounted for during the site selection process. There are several reasons why this is the case. One of them is that typical approaches to site selection are too reductive to accommodate the complex interrelationships of the factors associated with collaboration, such as the topology or configuration of the built environment. Systems approaches, such as the network analysis presented in this paper, make it possible to select a site based on desirable organizational outcomes, like cross-disciplinary scientific collaboration.

Another reason is that, in line with the traditional atomistic approach to site selection, little is done to understand how the construction of a new scientific facility is a systems intervention (the building) in an existing, complex system of systems (the campus). With its agnosticism about levels of analysis, networks analysis enables us to examine systems of systems. Thus, we can develop a better-informed sense of where to best locate new facilities so that we can optimize or maximize for the objectives that are critical to the bottom lines of higher education institutions. In the long run, given that it is more likely than not that we will build increasingly fewer new scientific facilities relative to the overall national expenditures on the research enterprise, future work could explore the cost-effectiveness of the network approach outlined in this study. It is well understood that it is important to select a site with favorable environmental conditions as this can reduce the need for costly future modifications and better ensure the sustainability of the facility. It is standard practice to include environmental considerations like natural light, air quality, and thermal conditions, all of which are essential for creating built environments that are conducive for research in fields such as the plant sciences. Just as important, however, is strategically locating scientific facilities to foster the multi- and inter-disciplinary science that higher education institutions are desperately seeking to stimulate with each increasingly diminishing opportunity to construct a new science building. This study suggests that it is possible to analyze potential sites for new scientific facilities in terms of their potential for scientific collaboration. This allows us to steward scarce resources in ways that are more cost-effective, environmentally sustainable and responsible, and likely to meet key organizational imperatives.

Aligning the site selection process with key organizational imperatives in higher education promotes the long-term viability of new scientific facilities. This is because, as alluded to earlier, facilities are less likely to need costly modifications or be torn down for not supporting the key outcomes of organizational imperatives like cross-disciplinary collaboration, factors such as talent attraction and retention. In this study it was straightforward to identify which of the two potential sites was better suited for the PESB at Michigan State University. Overall, site A’s network is less fragmented, more cohesive, and more efficient, making it better suited for fostering interactions and efficient movement across its spaces. In other words, site A is better suited for PESB given the university’s imperatives around scientific collaboration. But it is conceivable that there are potential instances where there may not be a clear-cut difference between two or more potential sites. Future studies could address these scenarios and focus on the development of more sophisticated metrics that can facilitate the adoption of the network approach for the site selection process.

## Acknowledgments

The author was solely responsible for all aspects of the manuscript’s development.
