# Correction: Network analysis and site selection for cross-disciplinary collaboration: The Plant and Environmental Science Building at Michigan State University

**DOI:** 10.1371/journal.pone.0339903

**Published:** 2025-12-29

**Authors:** Felichism Kabo

The Acknowledgement statement for this article is incorrect. The author would like to thank everyone at Michigan State University (MSU) and CannonDesign whose contributions helped make the study possible, especially: Charles Smith (CannonDesign), Dick Temple (MSU), Dave Dewitt (MSU), Alejandro Stochetti (CannonDesign), Alexandra Shinewald (CannonDesign), Amr Abdel-Azim (MSU), Scott Friend (MSU), Trevor Calarco (CannonDesign), and Stephanie Mamouzellos (CannonDesign).
